# Comparison of Laparoscopic Intraperitoneal Onlay Mesh (IPOM) Hernioplasty With Laparoscopic IPOM-Plus: Our Initial Experience in Pakistan

**DOI:** 10.7759/cureus.54007

**Published:** 2024-02-11

**Authors:** Fahad Yasin, Ali Abaid, Ahsan Shafiq, Muhammad Umar, Wasim Hayat Khan, Mahmood Ayyaz, Usman Ismat Butt

**Affiliations:** 1 Surgical Oncology, Shaukat Khanum Memorial Cancer Hospital and Research Centre, Lahore, PAK; 2 General Surgery, Shalamar Medical and Dental College, Lahore, PAK; 3 General Surgery, Services Hospital/Services Institue of Medical Sciences (SIMS), Lahore, PAK; 4 General Surgery, Sir Ganga Ram Hospital, Fatima Jinnah Medical University (FJMU), Lahore, PAK; 5 General Surgery, Mayo Hospital, King Edward Medical University (KEMU), Lahore, PAK

**Keywords:** ipom plus, ipom, laparoscopic, seroma, recurrence, fascial defect closure, ventral hernia

## Abstract

Objectives

Laparoscopic intraperitoneal onlay mesh hernioplasty (IPOM) for ventral hernias has been used for a long time. However, there have been some issues associated with it, thereby leading to the introduction of a new technique that involves laparoscopic closure of the fascial defect with suture followed by intraperitoneal onlay mesh placement (IPOM-Plus). We carried out this study to compare the outcome of laparoscopic IPOM with fascial defect closure versus without defect closure in midline ventral hernia repair in terms of recurrence.

Methodology

This comparative study was carried out in the Department of Surgery, Services Hospital, Lahore, from October 16, 2020, to April 15, 2022. A total of 84 patients of both genders, aged between 18 and 70 years, presenting with midline ventral hernia were included in the study. Patients with recurrent hernia, unstable cardiopulmonary conditions, neurological or psychiatric diseases, chronic renal disease, congestive cardiac failure, and chronic obstructive pulmonary disease (COPD) were excluded from the study. Patients were assigned to two groups. Group 1 underwent IPOM with the closure of the defect, and Group 2 underwent IPOM without the closure of the defect. Patients were observed for immediate postoperative complications. Patients were monitored for one year to assess recurrence through clinical evaluation and ultrasonography.

Results

In this study, seroma formation was found in 3 (7.14%) patients for laparoscopic IPOM with fascial defect closure and 10 (23.81%) in those undergoing laparoscopic IPOM without defect closure (*P*-value = 0.035). Recurrence was identified in 2 (4.76%) patients undergoing laparoscopic IPOM with fascial defect closure and 9 (21.43%) in those undergoing laparoscopic IPOM without defect closure (*P*-value = 0.024).

Conclusions

This study concluded that the frequency of recurrence is less after laparoscopic IPOM with fascial defect closure in midline ventral hernia repair than after laparoscopic IPOM without fascial defect closure.

## Introduction

Hernia is defined as an abnormal protrusion of an organ or tissue through a defect in its surrounding wall. A ventral hernia is a hernia that protrudes through the anterior abdominal wall fascia [1]. Midline ventral hernias include umbilical, paraumbilical, epigastric, and midline incisional hernias. Incisional hernias occur as a result of inadequate healing of a previous incision [1]. Abdominal wall hernias impart a significant burden on the healthcare system. A study published in 2019 showed a prevalence of 20.9% of abdominal wall hernias in the general population [2]. Umbilical and epigastric hernias constitute 10% of all abdominal hernias [1,2]. Incisional hernias have been reported in 10% to 50% of laparotomy incisions and 1% to 5% of laparoscopic port-site incisions [3].

The usual symptoms of patients with ventral hernia include pain, discomfort, and feeling of mass. The bowel may incarcerate or strangulate presenting with severe abdominal pain, fever, and obstructive signs and requiring emergency surgery. A detailed abdominal examination is usually sufficient to make a diagnosis. The most accurate radiographic diagnostic tool is computed tomography (CT). CT scans reveal the exact size and location of the abdominal wall defect, the degree of atrophy of the abdominal wall muscle around the defect site, and the relationship between the intraperitoneal organs and the hernia sac or abdominal wall defect [3].

Surgical repair of ventral hernias may be complicated by infection, seroma/hematoma formation, postoperative pain, recurrence, and longer stay at the hospital. The repair may be done by open or laparoscopic approach. Laparoscopic repair is associated with fewer postoperative complications, a lesser rate of recurrence, and a shorter stay in the hospital [4,5]. First reported by Karl Leblanc in 1993, the laparoscopic approach for ventral hernia repair has been accepted widely due to the better post-operative outcomes but there are still some issues related to a laparoscopic approach that cause controversy [6]. The laparoscopic repair involves bridging the defect from the peritoneal side with a mesh. This is known as the intraperitoneal onlay mesh (IPOM) repair. It is, however, associated with postoperative bulging or eventration of mesh, seromas, recurrences, and nonrestoration of abdominal muscle function [7]. To overcome these, a sutured closure of the defect in the fascia in addition to intraperitoneal mesh reinforcement, called IPOM-Plus repair, is now advised [8].

A few studies have been conducted to compare the outcomes after IPOM and IPOM-Plus with conflicting results. Furthermore, data on the comparison of the two techniques is scarce from Pakistan. This study aims to fill this knowledge gap. The objective of our study was to compare the outcome of laparoscopic IPOM with fascial defect closure versus without defect closure in midline ventral hernia repair in terms of recurrence and seroma formation.

## Materials and methods

We carried out a comparative study in the Department of Surgery, Services Hospital, Lahore, from October 16, 2020, to April 15, 2022. We calculated a sample size of 84 (42 in each group) using the WHO calculator, with a significance level of 5%, a study power of 80%, an anticipated incidence of seroma in IPOM with closure at 2.5%, and an incidence in IPOM without closure at 12.5% [9]. We employed nonprobability consecutive sampling, enrolling patients of both genders aged between 18 and 65 seeking management for midline ventral hernia. A patient was identified as having a ventral hernia if there was a protrusion through the anterior abdominal wall fascia in the midline, including umbilical, paraumbilical, epigastric, and midline incisional hernias. Patients with obstructed or strangulated hernias were excluded from this study, as emergency surgery is required in such cases and mesh placement is not performed in our setup under these circumstances. Additionally, individuals with a documented defect size of less than 3 cm on preoperative examination and ultrasound were excluded, as they are considered candidates for a simple repair. The study included patients classified as ASA 1 to 3, while those classified as ASA 4 and 5 were excluded.

The research proposal has been submitted to the Institutional Review Board (IRB) for approval. After obtaining approval from the IRB with reference letter no. IRB/2020/635/Services Institute of Medical Sciences dated April 13, 2020, informed consent was obtained from patients who met the inclusion/exclusion criteria and expressed willingness to participate in the study. This process was conducted at the outdoor departments of Services Hospital, Lahore. A detailed history was taken, and examinations were conducted for all patients. Data were entered in a proforma. Two groups were formed: Group 1 underwent IPOM with closure of the defect, and Group 2 underwent IPOM without closure of the defect, based on intra-operative findings as determined by the surgical team. These procedures were performed on an elective list. All the procedures were performed by the same surgical team. In these procedures, a closed pneumoperitoneum was established at Palmer’s point. Palmer's point is an area in the midclavicular line in the left upper quadrant a finger breadth below the costal margin. It is used to enter into the abdomen away from the midline. The primary port was inserted in the anterior axillary line at the level of the umbilicus, and two secondary ports were inserted in the mid-clavicular line. The general abdominal survey was conducted, and hernial contents were reduced. In Group 1, primary closure of the defect was done using proline 1/0 suture, while this step was skipped in Group 2 patients. Mesh was applied and fixed with the help of tackers in such a way that the mesh extends 5 cm further from the margins of the defect in every direction. Omentum was spread over the gut. Following the elimination of the pneumoperitoneum, layered closure in reverse was performed. Patients were then monitored for immediate postoperative complications, such as seroma formation. Stable patients were discharged upon meeting the established discharge criteria. Patients were followed for one year to assess recurrence through clinical evaluation and ultrasonography. All data were recorded in a proforma, including other variables such as mean hospital stay.

The collected information was analyzed by IBM SPSS Statistics for Windows, Version 21.0 (IBM Corp., Armonk, NY) software. Age, duration of disease, and body mass index (BMI) were presented as mean and standard deviation. Gender, type of hernia, presence of recurrence, and seroma formation were presented as frequency and percentage. The outcome was compared by chi-square test and a *P*-value ≤ 0.05 was considered significant. Stratification was done for age, gender, BMI, duration of disease, defect size, and type of hernia. Post-stratification, chi-square analysis was done to assess its effect on the outcome. A *P*-value ≤ 0.05 was considered significant.

## Results

The patient's and pathology characteristics are summarized in Table 1. The majority of the patients belonged to the third and fourth decades of life. Males outnumbered females slightly in this study. Most patients had symptoms for more than a year before undergoing surgery. More than two-thirds of the patients in our study had defects smaller than 5 cm, while the most common hernial type was umbilical.

**Table 1:** Patient and pathology characteristics (total number of cases, n = 84).

Characteristic	Group 1	Group 2	Overall
Age (Mean ± SD) ( in years)	39.52 ± 12.71	40.57 ± 13.70	40.27 ± 13.67
Gender (*n*, %)			
Male	25 (59.52%)	23 (54.76%)	48 (57.14%)
Female	17 (40.48%)	19 (45.24%)	36 (42.86%)
Duration of symptoms (years), *n* (%)			
<1 year	10 (23.81%)	07 (16.67%)	17 (20.24%)
>1 year	32 (76.19%)	35 (83.33%)	67 (79.76%)
Size, *n* (%)			
<5 cm	27 (64.29%)	25 (59.52%)	52 (61.90%)
>5 cm	15 (35.71%)	17 (40.48%)	32 (38.10%)
Body mass index (BMI), *n* (%)			
<30 kg/m^2^	16 (38.10%)	22 (52.38%)	38 (45.24%)
>30 kg/m^2^	26 (61.90%)	20 (47.62%)	46 (54.76%)
Type (number of cases, *n*, %)			
Epigastric	7 (16.67%)	5 (11.9%)	12 (14.29%)
Para-umbilical	17 (40.48%)	18 (42.86%)	35 (41.67%)
Umbilical	15 (35.71%)	17 (40.48%)	32 (38.10%)
Incisional	3 (7.14%)	2 (4.76%)	5 (5.95%)

We found that seroma formation was less in patients undergoing laparoscopic IPOM with fascial defect closure as compared to laparoscopic IPOM without defect closure (*P*-value = 0.035). Similarly, recurrence at the 1-year follow-up was also found to be lower in laparoscopic IPOM with fascial defect closure (*P*-value = 0.024) (Table 2).

**Table 2:** Comparison of outcome of laparoscopic IPOM with fascial defect closure versus without defect closure in midline ventral hernia repair (total number of cases, n = 84). *P*-value < 0.05 is taken as significant. IPOM, intraperitoneal onlay mesh

Outcome	Group 1 (*n* = 42), *n* (%)		Group 2 (*n* = 42), *n* (%)		*P*-value
	Yes	No	Yes	No	
Seroma formation	03 (7.14%)	39 (92.86%)	10 (23.81%)	32 (76.19%)	0.035
Recurrence	02 (4.76%)	40 (95.24%)	09 (21.43%)	33 (78.57%)	0.024

When stratification of characteristics was performed, it was observed that seroma formation was significantly reduced in patients undergoing laparoscopic IPOM-Plus who were younger than 45 years (*P *= 0.035), had a defect size less than 5 cm (*P *= 0.025), had a duration of symptoms less than one year (*P *= 0.023), or those who had a para-umbilical-type hernia (*P *= 0.009) (Table 3).

**Table 3:** Stratification of seroma formation with respect to age, gender, BMI, duration of disease, defect size, and type of hernia (total number of cases n = 84). *P*-value < 0.05 is taken as significant.

Confounders		Group 1 (*n* = 42), *n* (%)		Group 2 (*n* = 42), *n* (%)		*P*-value
		Seroma		Seroma		
		Yes	No	Yes	No	
Age (years)	18-45	01 (1.19%)	23 (27.38%)	06 (7.14%)	17 (20.23%)	0.035
	46-80	02 (2.38%)	16 (19.94%)	04 (4.76%)	15 (18.75%)	0.412
Gender	Male	03 (3.75%)	22 (27.5%)	07 (8.33%)	16 (19.04%)	0.116
	Female	00	17 (20.23%)	03 (3.57%)	16 (19.05%)	0.087
Duration (months)	≤12	00	10 (11.90%)	03 (3.57%)	04 (4.76%)	0.023
	>12	03 (3.57%)	29 (34.52%)	07 (8.33%)	28 (33.33%)	0.223
Size of the defect	≤5 cm	02 (2.38%)	25 (29.76%)	08 (9.52%)	17 (20.23%)	0.025
	>5 cm	01 (1.19%)	14 (16.66%)	02 (2.38%)	15 (17.85%)	0.621
Body mass index (BMI ) (kg/m^2^)	≤30	00	16 (19.05%)	04 (4.76%)	18 (21.42%)	0.071
	>30	03 (3.57%)	23 (27.3%)	06 (7.14%)	14 (16.66%)	0.118
Type	Epigastric	00	07 (8.33%)	01 (1.19%)	04 (4.76%)	0.217
	Para-umbilical	00	17 (20.23%)	06 (7.14%)	12 (14.28%)	0.009
	Umbilical	03 (3.57%)	12 (14.28%)	03 (3.57%)	14 (16.66%)	0.865
	Incisional	00	03 (3.57%)	00	02 (2.38%)	-

Similarly, after stratification, it was seen that recurrence at one year was significantly reduced in patients undergoing laparoscopic IPOM-Plus who were younger than 45 years (*P *= 0.003) (Table 4).

**Table 4:** Stratification of recurrence with respect to age, gender, BMI, duration of disease, defect size, and type of hernia (total number of cases, n = 84). *P*-value < 0.05 is taken as significant.

Confounders		Group 1 (*n* = 42), *n* (%)		Group 2 (*n* = 42), *n* (%)		*P*-value
		Recurrence		Recurrence		
		Yes	No	Yes	No	
Age (years)	18-45	00	24 (28.57%)	07 (8.33%)	16 (19.05%)	0.003
	46-80	02 (2.38%)	16 (19.05%)	02 (2.38%)	17 (20.23%)	0.954
Gender	Male	01 (1.19%)	24 (28.57%)	04 (4.76%)	19 (22.61%)	0.129
	Female	01 (1.19%)	16 (19.05%)	05 (5.95%)	14 (16.66%)	0.101
Duration (months)	≤12	00	10 (11.90%)	01 (1.19%)	06 (7.14%)	0.218
	>12	02 (2.38%)	30 (35.7%)	08 (9.52%)	27 (32.14%)	0.057
Size of the defect	≤5 cm	01 (1.19%)	26 (30.95%)	05 (5.95%)	20 (23.81%)	0.066
	>5 cm	01 (1.19%)	14 (16.66%)	04 (4.76%)	13 (15.47%)	0.190
Body mass index (BMI) (kg/m^2^)	≤30	01 (1.19%)	15 (17.85%)	05 (5.95%)	17 (20.23%)	0.169
	>30	01 (1.19%)	25 (29.76%)	04 (4.76%)	16 (19.05%)	0.081
Type	Epigastric	00	07 (8.33%)	00	05 (5.95%)	-
	Para-umbilical	01 (1.19%)	16 (19.05%)	03 (3.57%)	15 (17.85%)	0.316
	Umbilical	01 (1.19%)	14 (16.66%)	06 (7.14%)	11 (13.09%)	0.051
	Incisional	00	03 (3.57%)	00	02 (2.38%)	-

## Discussion

Ventral hernia is a major cause of functional impairment, abdominal pain, and bowel obstruction. The overall incidence of primary ventral hernia is estimated to be between 4% and 5% in the literature, and ventral incisional hernia rates vary from 35% to 60% within five years after laparotomy [7]. The laparoscopic technique for repairing ventral and incisional hernias is now well established. However, issues related to laparoscopic ventral hernia repair such as the high recurrence rate of hernias with large fascial defects in extremely obese patients and seroma formation still cause problems. To overcome these problems, laparoscopic fascial defect closure with IPOM reinforcement (IPOM-Plus) has been introduced in the past decade [8]. The guidelines from the Society of American Gastrointestinal and Endoscopic Surgeons currently defer decisions regarding IPOM or IPOM-Plus to the surgeon, citing insufficient high-quality research to establish the potential benefits of either. In contrast, the guidelines from the International EndoHernia Society recommend defect closure.

We conducted this study to compare the outcome of laparoscopic IPOM with fascial defect closure versus without defect closure in midline ventral hernia repair in terms of seroma formation and recurrence. Our results also demonstrated a significant reduction in both seroma formation and the incidence of recurrence at one year in the group of patients undergoing laparoscopic IPOM-Plus compared to the group undergoing IPOM. A study published in 2019 comprising 100 patients divided into two groups showed that patients with defect closure had a lesser rate of seroma formation (10% versus 18%). It showed a lesser rate of recurrence with defect closure, that is, 6% versus 18% (*P* = 0.07) in the case of all ventral hernias. It showed a significant reduction of recurrence rate in the closure of midline ventral hernias, that is, 5% versus 24% (*P *= 0.04) [9]. A recent systematic review of 3,638 patients concluded that IPOM-Plus was more effective than IPOM [10]. Seroma formation is much lower in IPOM-Plus as compared to IPOM (2.5% vs. 12.2%), but a multicenter study consisting of 1,594 patients showed that comparisons between both groups were negative for any significant statistical difference in terms of recurrence, seroma formation, surgical site infection (SSI), deep/organ space SSI, reoperation, and readmission [11].

Postoperative seroma can naturally evolve in different ways, namely, resorption, persistence, or complication. The risk factors for seroma were BMI (obesity), previous surgery, SSI, number of previous procedures, previous hernia repair, size of defect, and excessive use of cauterization. Moreno-Egea et al. [12] evaluated the size of the defect in 315 patients operated on for hernias between 5 and 15 cm in size. The size of the defect was an independent prognostic factor for recurrence with a threshold of 10 cm. We, however, were unable to find any significant correlation in this regard. However, there was a significant reduction in the seroma formation in patients with defect sizes smaller than 5 cm. Ali et al. reported a newer technique based on wound closure, which involved bridging the defect with a peritoneal bridge, which showed similar promising results as compared to IPOM-Plus [6].

Our study has several limitations. It is a single-center study with only a small number of patients. Additionally, the follow-up period was up to one year; a longer follow-up period would be necessary to assess the long-term outcomes of the procedures. We used ultrasound for recurrence evaluation because it is more cost-effective and easily available; however, this choice may have resulted in decreased detection of recurrence. In a recent randomized controlled trial involving eighty patients conducted by Christoffersen et al. [13], CT scans were utilized to assess seroma formation and recurrence. The study demonstrated that closure reduced the incidence of both, although the reported rates were notably higher. Still, our results showed that short-term outcomes with IPOM-Plus compared to IPOM are equal and better in some cases, especially in patients younger than 45 and when the defect size is less than 5 cm.

## Conclusions

This study concludes that the frequency of postoperative seroma formation and recurrence is lower after laparoscopic IPOM with fascial defect closure (IPOM-Plus) in midline ventral hernia repair compared to laparoscopic IPOM without fascial defect closure. This is particularly evident in patients under the age of 45, those with smaller-sized defects, para-umbilical hernia, and early disease diagnosis. However, larger-sized studies are needed before making any definitive recommendations.
